# Beyond the Curtains: Identification of the Genetic Cause of Foetal Developmental Abnormalities Through the Application of Molecular Autopsy

**DOI:** 10.3390/genes16101167

**Published:** 2025-10-02

**Authors:** Beatrice Spedicati, Giulia Pianigiani, Aurora Santin, Vanessa Rebecca Gasparini, Ilaria Falcomer, Agnese Feresin, Maria Teresa Bonati, Daniela Mazzà, Elisa Paccagnella, Domizia Pasquetti, Elisa Rubinato, Claudio Granata, Flora Maria Murru, Maurizio Pinamonti, Rossana Bussani, Ilaria Fantasia, Tamara Stampalija, Paolo Gasparini, Stefania Zampieri, Giorgia Girotto

**Affiliations:** 1 Department of Medicine, Surgery and Health Sciences, University of Trieste, 34149 Trieste, Italygiulia.pianigiani@burlo.trieste.it (G.P.); aurora.santin@burlo.trieste.it (A.S.); vanessarebecca.gasparini@burlo.trieste.it (V.R.G.); ilaria.falcomer@gmail.com (I.F.); feresin.agnese@gmail.com (A.F.); tamara.stampalija@burlo.trieste.it (T.S.); paolo.gasparini@burlo.trieste.it (P.G.); giorgia.girotto@burlo.trieste.it (G.G.); 2 Medical Genetics Unit, Institute for Maternal and Child Health—I.R.C.C.S. “Burlo Garofolo”, 34137 Trieste, Italy; mariateresa.bonati@burlo.trieste.it (M.T.B.); daniela.mazza@burlo.trieste.it (D.M.); elisa.paccagnella@burlo.trieste.it (E.P.); domizia.pasquetti@burlo.trieste.it (D.P.); elisa.rubinato@burlo.trieste.it (E.R.); 3 Radiology Department, Institute for Maternal and Child Health—I.R.C.C.S. “Burlo Garofolo”, 34137 Trieste, Italy; claudio.granata@burlo.trieste.it (C.G.); floramaria.murru@burlo.trieste.it (F.M.M.); 4 Department of Pathology, University of Trieste, 34149 Trieste, Italy; maurizio.pinamonti@asugi.sanita.fvg.it (M.P.); rossana.bussani@asugi.sanita.fvg.it (R.B.); 5 Unit of Fetal Medicine and Prenatal Diagnosis, Institute for Maternal and Child Health—I.R.C.C.S. “Burlo Garofolo”, 34137 Trieste, Italy; ilaria.fantasia@burlo.trieste.it

**Keywords:** foetal abnormalities, termination of pregnancy, Whole-Exome Sequencing, minigene splicing assay

## Abstract

**Background**: Foetal structural abnormalities can be detected in approximately 3% of all pregnancies and frequently remain without a genetic diagnosis. This study aims to apply an integrated approach with the final goal of providing a molecular diagnosis in the challenging Italian setting of early termination of pregnancy. **Methods**: In a cohort of 86 foetuses, *post-mortem* dysmorphological examination, radiological assessments, and molecular autopsy through Whole-Exome Sequencing—WES—analysis were performed. **Results**: Forty-two foetuses were phenotypically classified as presenting a single major malformation (i.e., central nervous system, skeletal, urogenital, or cardiac anomalies, or fluid accumulation), while 44 foetuses presented multiple malformations and/or dysmorphic features. Overall, WES provided a diagnostic yield of 26.7%; additionally, seven Variants of Uncertain Significance (VUS) potentially liked to the foetal phenotype were identified. The highest detection rate was achieved for foetuses presenting a single major urogenital (50%) or skeletal (42.9%) malformation, followed by foetuses presenting multiple malformations (27.3%). Peculiar results of particular interest were (1) the identification of two splicing variants (within the *INPPL1* and *RHOA* genes), functionally characterised through minigene assay, which contributed to evaluate their pathogenicity, and (2) the identification of a novel de novo missense *ZNF292* variant (NM_015021.3:c.6325A>C p.(Ser2109Arg)) in a foetus affected by corpus callosum hypoplasia. The *ZNF292* gene is associated with the Intellectual developmental disorder, autosomal dominant 64 and this finding represents the first report of prenatally detected anomalies of the central nervous system in a foetus carrying a *ZNF292* variant. **Conclusions**: This study underlines the diagnostic utility of an integrated approach to achieve a precise genetic diagnosis for structural foetal abnormalities, thus providing families with precise recurrence risk estimations and detailed options about future pregnancies. Additionally, a systematic implementation of this strategy could be crucial to better characterise new variants and discover new genes involved in embryonic and foetal development.

## 1. Introduction

Genetic disorders affect approximately 3.5–5.9% of the global population, and these estimates might be even higher when considering structural foetal abnormalities identified during pregnancy, as a variable proportion of these foetuses are miscarried, stillborn, or terminated [1,2,3]. The routine application of high-resolution ultrasonography in prenatal care has substantially improved the early detection and characterisation of foetal anomalies, refs. [4,5] which can occur as isolated phenomena, further classified as minor or major malformations, or present in broader syndromic patterns (multiple malformations) [6].

According to international guidelines, upon the detection of a foetal structural abnormality, pregnant women should be referred promptly for expert evaluation. This consists of a structured, detailed anatomic ultrasound that includes the evaluation of the entire foetal anatomy and size, cardiac activity, placental appearance and location, and amniotic fluid volume [7]. In specific cases, such as upon the detection of brain malformations, foetal Magnetic Resonance Imaging (MRI) is indicated to complement the ultrasound examination with the aim of confirming ultrasound findings or acquiring additional phenotypic information [8]. In order to pinpoint the exact aetiological cause of foetal abnormalities, invasive diagnostic testing is indicated and requires chorionic villi or amniotic fluid sampling, according to the gestational age [9]. Until the 2000s, prenatal diagnosis gold standard was karyotyping [10], whereas in the last two decades, chromosomal microarrays (CMAs) have become the first-line diagnostic test [11,12], as they detect smaller chromosomal imbalances. Overall, these techniques allow to identify a molecular diagnosis in approximately 40% of cases of foetal anomalies [4,13,14]. Thus, half of the cases with foetal abnormalities remain without a genetic diagnosis. Recently, several studies have proven the added clinical value of Next Generation Sequencing (NGS) technologies, such as Whole-Exome Sequencing (WES), in providing a better and more accurate prenatal genetic diagnosis. According to a recent systematic review and meta-analysis of studies focused both on ongoing pregnancies and pregnancies that underwent termination, WES analysis improves the diagnostic yield in an additional 31% of cases of foetal structural anomalies [13,15]. Specifically, it has been demonstrated that the diagnostic yield of WES differs significantly between different phenotypic groups, being the highest for single major malformations, such as skeletal alterations (53% [95% CI 42–63%]) and neuromuscular abnormalities (37% [20–54%]), and for multiple malformations (29% [22–35%]) [15]. Therefore, a correct interpretation of WES data relies on a detailed description of the foetal phenotype, which is often challenging to obtain during early foetal development due to (i) the ongoing and incomplete physiological maturation of tissues and organs, (ii) the impossibility of assessing functional alterations (i.e., intellectual, developmental, or sensory abilities), (iii) the incompleteness and non-specificity of pathological clinical features, and (iv) the limitation of tools for prenatal phenotype assessment [16,17]. All these difficulties could be further enhanced whenever the decision about continuation or termination of pregnancy needs to be made in the early pregnancy stages, as established by specific laws in different countries. In these cases, considering the uncertainty about the aetiological cause of foetal malformations and the recurrence risk for subsequent pregnancies, it is of paramount importance to offer additional diagnostic evaluations. To overcome such a complex scenario, there is a necessity to have a qualified multidisciplinary team (i.e., Medical Geneticists, Gynaecologists, Radiologists, Neonatologists, Pathologists) to provide the most accurate clinical characterisation in combination with highly sophisticated *post-mortem* analyses (i.e., phenotypic characterisation, foetal autopsy, WES, etc.) [16,17,18,19].

Here we report our experience in investigating 86 foetuses negative to CMA analysis that were fully characterised through *post-mortem* dysmorphological examination, radiological assessments, autopsy and molecular analyses. The aim of this study was to verify for the first time the detection rate of WES in the challenging Italian setting of early termination of pregnancy, with the final goal of elucidating the causes of foetal malformations and providing couples with information on future pregnancies recurrence risks.

## 2. Materials and Methods

### 2.1. Recruitment of Cases and Inclusion and Exclusion Criteria

Eighty-six foetuses presenting structural developmental abnormalities from pregnancies terminated between 2016 and 2024 were recruited by the Medical Genetics Unit of the I.R.C.C.S. “Burlo Garofolo” (Trieste, Italy). Only foetuses presenting a single major malformation or multiple congenital anomalies confirmed by *post-mortem* clinical investigations were included in the study. Foetuses presenting only minor malformations were excluded from the analysis, considering the low pre-test probability of providing a genetic diagnosis [20,21]. Only foetuses negative to Single Nucleotide Polymorphism (SNP) array analysis, which is currently employed in our laboratory as first-tier genetic testing in the prenatal setting, were considered for WES analysis (see Section 2.5 and Section 2.6).

### 2.2. Diagnostic Investigations Before Termination of Pregnancy

For each pregnancy, the clinical characterisation included first trimester ultrasonography to evaluate the nuchal translucency (NT) and assess the presence of soft markers [22]. In specific cases, pre-morphological ultrasound was performed at 16 weeks of gestation, while the second trimester scan, specifically aimed at evaluating foetal development and identifying any malformation [23], was performed between 18 and 21 + 6 weeks of gestation. If structural anomalies were detected, a second-level ultrasound was performed to provide a more detailed anatomical study. Additionally, in the presence of central nervous system (CNS) anomalies, pregnant women were also referred for foetal MRI [24]. Whenever foetal malformations were detected, genetic counselling was offered. A three-generation familial anamnesis was collected to identify other family members affected by a genetic condition and to verify the possible presence of a consanguineous relationship between the parents. Furthermore, particular attention was paid to the ongoing pregnancy history, with the aim of better clarifying parental age and mode of conception and identifying possible risk factors, as maternal diseases, teratogens exposure, medications or infections. During genetic counselling, invasive prenatal procedures, including chorionic villus sampling or amniocentesis, accordingly to gestational age, were discussed and first-tier genetic testing (i.e., Quantitative Fluorescence Polymerase Chain Reaction (QF-PCR) and SNP-array analyses) were offered. Whenever these analyses resulted negative, a multidisciplinary consultation that included Medical Geneticists and Gynaecologists, and, based on the specifically highlighted anomalies, Radiologists, Neonatologists, Paediatric surgeons, Cardiologists, Neurologists, Nephrologists, or Pneumologists, was performed in order to inform couples on the clinical implications of the identified morphological abnormalities. Couples were therefore offered the possibility to continue or terminate the pregnancy on the basis of the foetal phenotype, according to the Italian law 194/78 on termination of pregnancy.

### 2.3. Diagnostic Investigations After Termination of Pregnancy

After termination of pregnancy, additional clinical investigations were performed whenever possible and according to parental consent. Briefly, a comprehensive dysmorphological examination was conducted to provide detailed somatometric measurements and to identify any facial dysmorphic features, skeletal, ectodermal, and genital anomalies [25]. Photographic records were obtained and, subsequently, radiological exams were performed (anteroposterior and lateral radiographs of the foetus, total-body computed tomography scan (CT), and total-body MRI). Afterwards, foetuses were sent for autopsy, where a thorough study of the internal organs was performed, according to international guidelines [26].

### 2.4. DNA Extraction and Quality Control

Foetal genomic DNA was extracted from either chorionic villi or amniotic fluid obtained during pregnancy or skin specimens obtained after termination, while parental DNA was extracted from peripheral blood according to standard protocol (QIAsymphony Midi Kit, Qiagen, Hilden, Germany). DNA concentration was measured using the Qubit DNA Broad Range kit (Thermo Fisher Scientific, Waltham, MA, USA). QF-PCR on both foetal and maternal genomic DNA (Thermo Fisher Scientific, Waltham, MA, USA) was used to prevent misdiagnosis due to maternal cell contamination.

### 2.5. SNP-Array Analysis

SNP-array analysis was performed using the Infinium CytoSNP-850K BeadChip (Illumina Inc., San Diego, CA, USA). Genomic DNA for each sample was processed according to Illumina’s Infinium HD Assay Super protocol. Raw image intensity data normalization, genotype clustering, and genotype calling of individual samples were performed using Illumina’s Genome Studio v2.0.5 software (cnvpartition 3.2.1). Copy Number Variations (CNVs) were mapped to the hg19 human reference genome and annotated with UCSC RefGene. Allele detection and genotype calling were performed with Genome Studio v2.0.5 software, and data analysis was performed with NxClinical software version 6.1 (BioDiscovery, Inc., El Segundo, CA, USA). SNP-array analysis provides an average resolution of 50 Kb and a threshold of ten DNA probes is required to support CNVs reporting. Mosaicism detection cut-off was set at 10–20%, as per literature data [27].

### 2.6. WES Analysis and Data Interpretation

Trio WES analysis was carried out in the vast majority of cases. Whenever needed, the analysis was extended to other family members and, in three cases, the lack of one parent forced us to run WES only in duo. In all cases, WES was performed on an Illumina NextSeq 550 instrument (Illumina, San Diego, CA, USA); genomic libraries were prepared using the Twist Human Exome 2.0 Plus Comprehensive Exome Spike-in kit (Twist Bioscience, San Francisco, CA, USA), according to the manufacturer’s protocol. WES protocol and secondary and tertiary analyses were carried out as already reported [28]. Briefly, raw fastq files were processed using EnGenome Germline Pipeline (v2.3; enGenome, Pavia, Italy). Alignment to the human reference genome (hg19) was performed through BWA-mem algorithm [29] and variant calling was carried out for each sample with GATK HaplotypeCaller. Variant annotation and classification were conducted using the eVai software (v2.2-v3.1; enGenome, Pavia, Italy). WES data showed a mean on-target depth of 75.7Xa median on-target depth of 75.6X, with 97.8% of targets covered at 10X and 97.1% at 20X. Capture uniformity, callable exome size and contamination checks were assessed based on the manufacturer’s output data and quality control metrics.

Phenotypic filtering of variants was based on the clinical description of foetuses obtained with Human Phenotype Ontology (HPO) terms (https://hpo.jax.org/). Variant frequency was verified in NCBI dbSNP build 156 (https://www.ncbi.nlm.nih.gov/snp/) and gnomAD (https://gnomad.broadinstitute.org/). The deleteriousness of all identified variants was assessed through in silico prediction tools [30,31,32,33,34]. SNVs leading to synonymous amino acid substitutions not predicted as damaging, not affecting splicing or highly conserved residues, and variants with a quality score (QUAL) < 20 or called in off-target regions were excluded. Pathogenicity of already-reported variants was assessed through ClinVar (https://www.ncbi.nlm.nih.gov/clinvar/) and The Human Gene Mutation Database (HGMD) Professional (https://my.qiagendigitalinsights.com/bbp/view/hgmd/pro/start.php). Variant classification, based on the American College of Medical Genetics and Genomics/Association for Molecular Pathology (ACMG/AMP) criteria [35], was carried out employing the variant interpretation platforms VarSome (https://varsome.com/) and Franklin (https://franklin.genoox.com/clinical-db/home), followed by manual curation. All databases have been last accessed on 31 December 2024.

Genes located within a CNV were evaluated for clinical relevance when information on dosage sensitivity (haploinsufficiency or triplosensitivity) was available according to literature data. Additionally, CNVs were assessed in detail whenever the suspected deletion or duplication involved genes associated with the foetal clinical phenotype.

All variants of interest were firstly deeply discussed to assess whether they could match the foetal phenotype and were confirmed by Sanger sequencing both in foetal and parental DNA. Whenever necessary, familial segregation of identified variants in additional family members was also carried out. Parental data from trio sequencing were used to confirm parentage and to establish whether the identified variants occurred de novo. In case of duo sequencing, the inheritance pattern of the detected variants was reported as assumed. The variant allele frequency (alternate/reference) of de novo variants ranged from 17% to 60%. Post-test genetic counselling was offered to all couples who underwent WES analysis to discuss the final results (positive, Variants of Uncertain Significance (VUS), and negative cases).

### 2.7. Minigene Assays

The potential effect of splice-site variants was performed through in silico analysis by the SpliceAI prediction tool (https://spliceailookup.broadinstitute.org (accessed on 31 December 2024)) [36]. To confirm these results, splicing minigene assays were performed to functionally characterise NM_001664.4(*RHOA*):c.408+2C>G and NM_001567.4(*INPPL1*):c.1497+5G>C variants. The exon and its adjacent intronic sequences containing wild-type or mutant splice-site variants were amplified from patients’ genomic DNA using KAPA HiFi HotStart ReadyMix (Roche Diagnostics, Almere, the Netherlands) and assembled into the XhoI/NotI-digested Exontrap pET01 vector (MoBiTec GmbH, Goettingen, Germany) using the In-Fusion HD Cloning Kit (Takara, Dalian, China). Before transfection, all constructs were verified by Sanger sequencing.

### 2.8. Cell Culture and Transfection

Human Embryonic Kidney (HEK293T) cells (ATCC, CRL-3216) were grown in Dulbecco’s modified eagle medium (DMEM) with L-glutamine (EuroClone, Milan, Italy) supplemented with 100 U/mL penicillin, 0.1 mg/mL streptomycin and 10% fetal bovine serum (EuroClone, Milan, Italy) at 37 °C with 5% CO_2_. For transfection, HEK293T cells were seeded into a 6-well plate (0.5 × 10^6^ cells/well) and transfected after 24 h using the Lipofectamine 2000 Transfection Reagent and 1.0 μg of each minigene diluted in Opti-MEM Reduced Serum Medium (Thermo Fisher Scientific, Waltham, MA, USA). All minigene transfection experiments were carried out in triplicate.

### 2.9. RNA Extraction and Transcript Analysis

Total RNA was extracted 24 h after transfection, using the Quick-RNA Miniprep Kit (Zymo Research, Irvine, CA, USA) and treated with DNase I, Amplification Grade (Thermo Fisher Scientific, Waltham, MA, USA). RNA concentration and quality was assessed using the NanoDrop One spectrophotometer. Reverse transcription was carried out with 500 ng of total RNA using the SensiFAST cDNA Synthesis Kit (Bioline|Meridian Bioscience, Cincinnati, OH, USA). cDNA was amplified using the GoTaq DNA Polymerase (Promega, Madison, WI, USA) and a vector-specific primer pair (Supplementary Table S1). Reverse Transcription Polymerase Chain Reaction (RT-PCR) products were separated by electrophoresis on 2% agarose gel, and Sanger sequencing of gel-extracted RT-PCR bands was performed.

## 3. Results

### 3.1. Demographic and Pregnancies Data

Eighty-six foetuses from 80 unrelated families presenting structural developmental abnormalities were enrolled in the study.

Maternal median age at enrolment was 33.4 ± 5.7 years. Six out of 86 pregnancies (7%) were terminated in the first trimester (gestational age 12.3 ± 0.3 weeks) by aspiration, while 80 pregnancies (93%) were interrupted in the second trimester (gestational age 20.6 ± 2.2 weeks) through a medical induction of labour. Twenty-two women (27.5%) reported previous foetal or newborn loss (Supplementary Figure S1), but none of them was investigated at genetic level.

In four cases, two foetuses conceived by the same couple were enrolled in the study, and in one case, three foetuses conceived from the same parents were recruited. Two pregnancies (2.3%) had been obtained through Assisted Reproductive Technologies, namely one case of heterologous (egg donation) and one case of homologous in vitro fertilisation.

### 3.2. Phenotypic Classification of Foetuses

Among the 86 recruited foetuses, 51 were males and 35 females. Phenotypic data was obtained by prenatal imaging reports (ultrasonography and MRI), dysmorphological examination after termination, *post-mortem* imaging (radiographs, CT scan, and MRI) and foetal autopsy. Notably, foetal autopsy was concordant with prenatal imaging in all cases, being able, for some foetuses, to further delineate the clinical features (Supplementary Table S2). These evaluations allowed to categorise foetuses into two main diagnostic groups, namely (i) “Single major malformation” and (ii) “Multiple malformations”. All foetuses belonging to the first category were subdivided according to the main affected organ or system into the following phenotypic groups: CNS anomalies, skeletal anomalies, urogenital anomalies, cardiac anomalies, and fluid accumulation. Foetuses belonging to the second group showed alterations in at least two different organs and/or dysmorphic features and were hypothesised to be affected by a multisystemic condition. A summary of the phenotypical features of the cohort is reported in Table 1, and detailed phenotypic features of all foetuses are reported in Supplementary Tables S2 and S3.

The “Single major malformation” group included 42 foetuses. Among them, the most frequent anomalies were those of the CNS, which included both isolated malformations, as abnormalities of the corpus callosum and the posterior cranial fossa, and complex malformations, as holoprosencephaly. All CNS anomalies were detected in the second trimester of pregnancy. Skeletal anomalies involving multiple bone segments could be detected earlier than CNS abnormalities, leading in some cases to first trimester termination. A minority of foetuses presented cardiac anomalies: three of them showed a hypoplastic left heart, one a coarctation of the aorta, and one multiple rhabdomyomas. Among cases displaying urogenital anomalies, two sibling foetuses presented bilateral renal agenesis, while one foetus presented unilateral renal agenesis and bladder hypoplasia, and the last foetus showed bladder exstrophy. Finally, a small proportion of foetuses presented fluid accumulation, both as increased NT and hydrops. Forty-four foetuses presented multiple malformations and/or dysmorphic features highlighted upon *post-mortem* dysmorphological examination that hinted at the presence of an underlying syndromic condition. In all phenotypic categories, there was no statistically significant difference in the frequency of malformations between male and female foetuses.

### 3.3. Molecular Autopsy Results

WES analysis, guided by the detailed clinical characterisation obtained through prenatal and *post-mortem* evaluations, allowed to achieve an overall diagnostic yield of 26.7% (23/86 foetuses; Table 2, Section A), with the identification of 24 variants in 20 genes classified as pathogenic or likely pathogenic according to ACMG/AMP guidelines [35]. Among them, the NM_000308.4(*CTSA*):c.990dupC p.(Cys331Leufs*56) variant has been identified in compound heterozygosity with the NM_000308.4(*CTSA*):c.753_755del p.(Asn251del) variant, that at present is classified as VUS; considering that *CTSA* is associated with Galactosialidosis (MIM: # 256540), which is an autosomal recessive disease, we deemed this finding as disease-causing [35]. Additionally, seven variants potentially linked to the foetal phenotype were classified as VUS (Table 2, Section B). It is worth noting that 3/7 VUS have already been reported in the scientific literature as disease-associated. Indeed, the *NOTCH2* variant NM_024408.4:c.5177G>A p.(Arg1726His) was previously described in a patient affected by congenital heart disease [37] and both the *COL6A1* variants NM_001848.3:c.751G>A p.(Glu251Lys) and NM_001848.3:c.1712A>C p.(Lys571Thr) were associated with neuromuscular disorder [38,39]. Finally, the minigene assay was employed to understand the functional effect of NM_001664.4(*RHOA*):c.408+2C>G and NM_001567.4(*INPPL1*):c.1497+5G>C variants (see Section 3.4).

Overall, a slightly higher diagnostic rate was achieved in the “Multiple malformations” group (12/44 solved cases, 27.3%) in comparison with the “Single major malformation” group (11/42 solved cases, 26.2%). In particular, within the “Single major malformation” group, the highest diagnostic yield was highlighted for urogenital (2/4, 50%) and skeletal anomalies (3/7, 42.9%). Additionally, a definitive genetic diagnosis was achieved in 1/5 fluid accumulation cases (20%), in 5/21 foetuses presenting CNS anomalies (23.8%), and in none of the foetuses presenting cardiac anomalies.

Concerning genotype–phenotype correlations, more than half of the highlighted variants were detected in genes already associated with non-syndromic conditions with well-characterised prenatal phenotypes. For instance, six different variants were identified in genes involved in CNS development and known to cause prenatally detectable brain malformations, such as *TUBA1A*, *ZIC2*, *ASPM*, *FOXG1*, and *ARX*. Additionally, biallelic variants were detected in the *CTSA* and *PEX1* genes, associated with severe metabolic disorders that often present with non-immune foetal hydrops. Four variants were highlighted in four genes known to cause skeletal dysplasias, namely *EBP*, *INPPL1*, *COL1A2*, and *SLC26A2*, being the last two characterised by high in utero lethality. Finally, one variant was identified in two related foetuses in the *GREB1L* gene, associated with renal agenesis, while in two other related cases, showing congenital heart malformations, a variant was identified in the *NOTCH2* gene.

**Figure 1:**
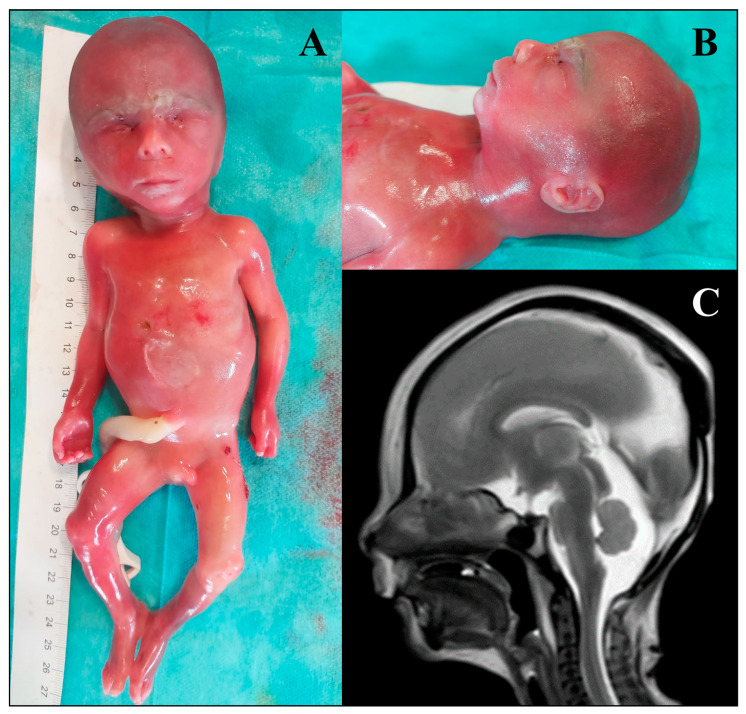
Phenotypic features of the foetus harbouring a heterozygous de novo variant in the *ZNF292* gene. (**A**) Overall foetal appearance: total body length: 26 cm; crown–rump length: 16 cm; head circumference: 19.2 cm; thoracic circumference: 16.8 cm; right foot sandal gap; normal male genitalia. (**B**) Left facial profile: mild microretrognathia. (**C**) foetal brain MRI: hypoplasia of the corpus callosum.

Furthermore, several foetuses presented deleterious variants in genes associated with syndromic conditions that are not usually characterised by an increased prenatal lethality and have been deeply studied in the postnatal setting, as *ARID1B*, *MECOM*, *FGFR2*, *NAA10*, *COL6A1*, *RPS19*, *TBX1*, *HRAS*, and *KMT2D*. Finally, a peculiar finding of this study is represented by the identification of a novel missense variant (NM_015021.3:c.6325A>C p.(Ser2109Arg)) in the *ZNF292* gene, whose pathogenic variants cause Intellectual developmental disorder, autosomal dominant 64 (MIM: # 619188), an autosomal dominant neurodevelopmental disorder. The variant is predicted as deleterious by all in silico software employed during the analysis (CADD score: 25.5; PaPI score: 0.928; PolyPhen2: 0.795; SIFT: 0.01) and the genetic intolerance profile for the protein domains assessed with the MetaDome web server (https://stuart.radboudumc.nl/metadome/ (accessed on 8 September 2025)) showed how the 1209 residue is intolerant to missense variants (tolerance score: 0.5). At present, only postnatal cases have been reported in the scientific literature [40], thus, our finding might implement the phenotypic characterisation of the disease, adding specific prenatal manifestations and possibly representing an early prenatal diagnosis (Figure 1).

As regards the pattern of inheritance, in accordance with literature data [19], the majority of variants (12/31–38.7%) occurred de novo in 11 different genes already known to be associated with autosomal dominant disorders. The *ARID1B* gene, associated with Coffin-Siris syndrome 1 (MIM: # 135900), and the *TUBA1A* gene, causative of Lissencephaly 3 (MIM: # 611603), were recurrently mutated. This supports previously published literature data that recognises how they are among the genes with the highest proportion of de novo mutation rates, both as an absolute occurrence and in consideration of gene size [41]. Conversely, in three other cases, inheritance from an unaffected parent, due to incomplete penetrance, was observed: a *TBX1* known heterozygous missense variant [42] in one foetus presenting with truncus arteriosus and interventricular defect, and an already reported *NOTCH2* heterozygous missense variant [37] in two foetuses conceived by the same couple presenting with a complex heart malformation and increased NT. Interestingly, the father of these last two foetuses reported to have had a sister, deceased in her second day of life due to a complex congenital heart malformation. Unfortunately, due to unavailability of her biological sample, it was not possible to perform the segregation analysis.

Additionally, a heterozygous novel variant in the *COL1A2* gene was assumed to have originated de novo in a foetus conceived through egg donation showing clinical features consistent with skeletal dysplasia (i.e., severe hypoplasia of the upper and lower limbs, short ribs and thoracic hypoplasia, trigonocephaly and metopic craniosynostosis). Finally, most likely, a familial segregation characterises a variant identified, in two sibling foetuses, in the *GREB1L* gene which causes Renal hypodysplasia/aplasia 3 (MIM: # 617805). The variant is assumed to have been inherited from the father, who showed unilateral renal agenesis, like the other fifteen-year-old son of the couple. The latter has been confirmed to also be a carrier of the variant, whereas, unfortunately, it was not possible to collect any biological paternal specimen and confirmatory segregation studies were not carried out. Furthermore, two de novo variants were identified in two female foetuses in genes associated with X-linked conditions, namely *EBP* and *ARX*; additionally, in six cases, homozygous or compound heterozygous variants were identified in genes associated with autosomal recessive conditions, namely *ASPM*, *INPPL1*, *CTSA*, *SLC26A2*, *COL6A1*, and *PEX1*.

A not fully conclusive diagnosis that represented a challenge in addressing family counselling was highlighted in (a) a female foetus presenting with clinical features consistent with skeletal dysplasia (i.e., hypoplasia and curved appearance of the upper and lower limbs, curved appearance of the ribs with thoracic hypoplasia and barrel chest) harbouring a maternally inherited known pathogenic variant in the *TRIP11* gene, associated with autosomal recessive Achondrogenesis, type IA (MIM: # 200600), and (b) a female foetus presenting with left diaphragmatic hernia and dysmorphic features carrying a germline variant, classified as a VUS, in the *RHOA* gene, which has been so far described only as somatically mutated in postnatal cases [43].

**Table 2:** Variants identified through WES analysis. Section A: cases solved through the identification of pathogenic or likely pathogenic variants. Section B: cases where a Variant of Uncertain Significance in a potentially causative gene was identified. Foetal ID: progressive identification number of foetuses enrolled in the study. Diagnostic group: main diagnostic category according to the presence of major malformations of a single organ or multiple anomalies, including facial dysmorphisms. Phenotypic group: specific anomalies divided by affected organ or hypothesis of syndromic involvement. Gene: list of genes carrying the identified variants. Associated disease and inheritance pattern: list of disorders associated with each gene according to OMIM^®^—Online Mendelian Inheritance in Man^®^; AD: autosomal dominant; AR: autosomal recessive; XLD: X-linked dominant; XL: X-linked. Variant: variant description according to the Human Genome Variation Society (HGVS) nomenclature guidelines; the NCBI RefSeq accession number of the considered protein-coding transcripts (NM_) is reported. Frequency: variant frequency is reported according to gnomAD v4. Zygosity: HET: the variant affects only one allele; HOM: the same variant is present on both alleles; COMP HET: two different variants are present on each allele. Inheritance: inheritance pattern of every identified variant established after parental segregation. ACMG/AMP classification: variants pathogenicity according to ACMG/AMP guidelines and applied criteria. Ref: references of publications reporting each variant. NA: Not Available.

**Section A: Variants Identified in Solved Cases**										
**Foetal ID**	**Diagnostic Group**	**Phenotypic Group**	**Gene**	**Associated** **Disease and Inheritance Pattern**	**Variant**	**Frequency**	**Zygosity**	**Inheritance**	**ACMG/AMP** **Classification**	**Ref.**
1	Single major malformation	CNS anomalies	*TUBA1A*	Lissencephaly 3 (MIM: # 611603) AD	NM_006009.3:c.521C>T p.(Ala174Val)	NA	HET	De novo	Likely Pathogenic (PS2, PM1, PM2_Supporting, PM5)	[44]
2	Single major malformation	CNS anomalies	*TUBA1A*	Lissencephaly 3 (MIM: # 611603) AD	NM_006009.3:c.190C>T p.(Arg64Trp)	0.00003965	HET	De novo	Pathogenic (PS2; PS3; PM1; PM2_supporting; PM5; PP3_moderate)	[19]
3	Single major malformation	CNS anomalies	*ZIC2*	Holoprosencephaly 5 (MIM: # 609637) AD	NM_007129.5:c.215delG p.(Gly72Ala fs*146)	NA	HET	De novo	Pathogenic (PVS1; PS2; PM2_supporting)	NA
4	Single major malformation	CNS anomalies	*ASPM*	Microcephaly 5, primary, autosomal recessive (MIM: # 608716) AR	NM_018136.5:c.7551T>A p.(Tyr2517*)	0.000002481	COMP HET	Maternal	Pathogenic (PVS1; PM2_supporting; PM3)	NA
					NM_018136.5:c.9926delG p.(Ser3309Ile fs*31)	6.198 × 10^−7^		Paternal	Pathogenic (PVS1; PM2_supporting; PM3)	NA
5	Single major malformation	CNS anomalies	*FOXG1*	Rett syndrome, congenital variant (MIM: # 613454) AD	NM_005249.5:c.256delC p.(Gln86Arg fs*106)	NA	HET	De novo	Pathogenic (PVS1; PS2_very strong; PM2_supporting)	[45]
6	Single major malformation	Skeletal anomalies	*COL1A2*	Osteogenesis imperfecta, type II (MIM: # 166210) AD	NM_000089.3:c.3125G>T p.(Gly1042Val)	NA	HET	*Assumed* de novo	Likely Pathogenic (PM1; PM2_supporting; PM5; PM6; PP3)	NA
7	Single major malformation	Skeletal anomalies	*EBP*	Chondrodysplasia punctata, X-linked dominant (MIM: # 302960) XLD	NM_006579.2:c.364G>A p.(Glu122Lys)	NA	HET	De novo	Pathogenic (PS2; PM1; PM2_supporting; PP3_strong)	[46]
8	Single major malformation	Skeletal anomalies	*TRIP11*	Achondrogenesis, type IA (MIM: # 200600) AR	NM_004239.3:c.673C>T p.(Arg225*)	0.000008070	HET	Maternal	Likely Pathogenic (PVS1; PM2_supporting)	[47]
9	Single major malformation	Urogenital anomalies	*GREB1L*	Renal hypodysplasia/ aplasia 3 (MIM: # 617805) AD	NM_001142966.3:c.3007dupA p.(Thr1003Asn fs*14)	NA	HET	Assumed paternal	Likely Pathogenic (PVS1; PM2_supporting)	NA
10	Single major malformation	Urogenital anomalies	*GREB1L*	Renal hypodysplasia/ aplasia 3 (MIM: # 617805) AD	NM_001142966.3:c.3007dupA p.(Thr1003Asn fs*14)	NA	HET	Assumed paternal	Likely Pathogenic (PVS1; PM2_supporting)	NA
11	Single major malformation	Fluid accumulation	*CTSA*	Galactosialidosis (MIM: # 256540) AR	NM_000308.4:c.990dupC p.(Cys331Leu fs*56)	0.000003099	COMP HET	Maternal	Likely Pathogenic (PVS1; PM2_supporting)	[48]
					NM_000308.4:c.753_755 del p.(Asn251del)	NA		Paternal	Variant of Uncertain Significance (PM2_supporting; PM3; PM4)	NA
12	Multiple malformations	Multisystemic condition	*ARID1B*	Coffin-Siris syndrome 1 (MIM: # 135900) AD	NM_001374820.1:c.4359G>A p.(Pro1453Pro)	6.258 × 10^−7^	HET	De novo	Likely Pathogenic (PS2; PM2_supporting; PP3)	[49]
13	Multiple malformations	Multisystemic condition	*ARID1B*	Coffin-Siris syndrome 1 (MIM: # 135900) AD	NM_001374820.1:c.1293_1314 del p.(Gly434Metfs*11)	NA	HET	De novo	Pathogenic (PVS1; PS2; PS4_moderate; PM2_supporting)	NA
14	Multiple malformations	Multisystemic condition	*MECOM*	Radioulnar synostosis with amegakaryocytic thrombocytopenia 2 (MIM: # 616738) AD	NM_001105077.3:c.2005C>T p.(Arg669*)	NA	HET	De novo	Pathogenic (PVS1; PS2; PM2_supporting)	NA
15	Multiple malformations	Multisystemic condition	*SLC26A2*	Achondrogenesis Ib (MIM: # 600972) AR	NM_000112.4:c.1336A>T p.(Lys446*)	0.000002478	HOM	Maternal and paternal	Pathogenic (PVS1; PM2_supporting; PM3_supporting)	NA
16	Multiple malformations	Multisystemic condition	*ARX*	Lissencephaly, X-linked 2 (MIM: # 300215) XL	NM_139058.3:c.206delA p.(Lys69fs*99)	NA	HET	De novo	Pathogenic (PVS1; PS2; PM2_supporting)	NA
17	Multiple malformations	Multisystemic condition	*FGFR2*	Apert syndrome (MIM: # 101200) AD	NM_000141.5:c.755C>G p.(Ser252Trp)	0.00004030	HET	De novo	Pathogenic (PS2; PS3; PM1; PM2_supporting; PM5_moderate)	[50]
18	Multiple malformations	Multisystemic condition	*NAA10*	Ogden syndrome (MIM: # 300855) XLD	NM_003491.4:c.92A>G p.(Tyr31Cys)	NA	HET	De novo	Likely Pathogenic (PS2; PS4_moderate; PM1_supporting; PM2_supporting)	[51]
19	Multiple malformations	Multisystemic condition	*RPS19*	Diamond-Blackfan anemia 1 (MIM: # 105650) AD	NM_001022.4:c.185G>A p.(Arg62Gln)	NA	HET	De novo	Pathogenic (PS2; PS3_supporting; PS4; PM1; PM2_supporting; PM5_strong; PP3_moderate)	[52]
20	Multiple malformations	Multisystemic condition	*TBX1*	Velocardiofacial syndrome (MIM: # 192430) AD	NM_080647.1:c.698C>T p.(Ser233Phe)	NA	HET	Maternal	Likely Pathogenic (PM1; PM2_supporting; PM5_supporting; PP3_strong)	[42]
21	Multiple malformations	Multisystemic condition	*HRAS*	Costello syndrome (MIM: # 218040) AD	NM_005343.4:c.37G>T p.(Gly13Cys)	NA	HET	De novo	Pathogenic (PS2_very strong; PS4; PM1; PM2_supporting; PM5_strong; PP3_moderate)	NA
22	Multiple malformations	Multisystemic condition	*KMT2D*	Kabuki syndrome 1 (MIM: # 147920) AD	NM_003482.4:c.643_644delCCinsTG p.(Pro215*)	NA	HET	De novo	Pathogenic (PVS1; PS2; PM2_supporting)	NA
23	Multiple malformations	Multisystemic condition	*PEX1*	Peroxisome biogenesis disorder 1A (Zellweger) (MIM: # 214100) AR	NM_000466.3:c.2760delA p.(Ala921Leu fs*40)	6.214 × 10^−7^	HOM	Maternal and paternal	Pathogenic (PVS1; PM2_supporting; PM3)	[53]
**Section B: Identification of Variants of Uncertain Significance**										
**Foetal ID**	**Diagnostic Group**	**Phenotypic Group**	**Gene**	**Associated** **Disease and Inheritance Pattern**	**DNA Change**	**Frequency**	**Zygosity**	**Inheritance**	**ACMG/AMP** **Classification**	**Ref.**
24	Single major malformation	CNS anomalies	*ZNF292*	Intellectual developmental disorder, autosomal dominant 64 (MIM: # 619188) AD	NM_015021.3:c.6325A>C p.(Ser2109Arg)	NA	HET	De novo	Variant of Uncertain Significance (PS2; PM2_supporting)	NA
25	Single major malformation	Skeletal anomalies	*INPPL1*	Opsismodysplasia (MIM: # 258480) AR	NM_001567.4:c.1497+5G>C	0.00001371	HOM	Maternal and paternal	Variant of Uncertain Significance (PM2_supporting; PM3_supporting; PP3)	NA
26	Single major malformation	Cardiac anomalies	*NOTCH2*	Alagille syndrome 2 (MIM: # 610205) AD	NM_024408.4:c.5177G>A p.(Arg1726His)	NA	HET	Paternal	Variant of Uncertain Significance (PM2_supporting)	[37]
27	Single major malformation	Fluid accumulation	*NOTCH2*	Alagille syndrome 2 (MIM: # 610205) AD	NM_024408.4:c.5177G>A p.(Arg1726His)	NA	HET	Paternal	Variant of Uncertain Significance (PM2_supporting)	[37]
28	Multiple malformations	Multisystemic condition	*RHOA*	Ectodermal dysplasia with facial dysmorphism and acral, ocular, and brain anomalies (MIM: # 618727) Somatic mosaicism	NM_001664.4:c.408+2C>G	NA	HET	De novo	Variant of Uncertain Significance (PS2; PM2_supporting; PP3)	NA
29	Multiple malformations	Multisystemic condition	*COL6A1*	Ullrich congenital muscular dystrophy 1A (MIM: # 254090) AR	NM_001848.3:c.1712A>C p.(Lys571Thr)	0.0002714	COMP HET	Maternal	Variant of Uncertain Significance (PM2_supporting; PP3_moderate)	[38]
					NM_001848.3:c.751G>A p.(Glu251Lys)	0.0002554		Paternal	Variant of Uncertain Significance (PM2_supporting; PP3)	[39]

### 3.4. Functional Validation of INPPL1 and RHOA Splicing Variants Through Minigene Assay

#### 3.4.1. Minigene Splicing Assay of NM_001567.4(*INPPL1*):c.1497+5G>C Variant

**Figure 2:**
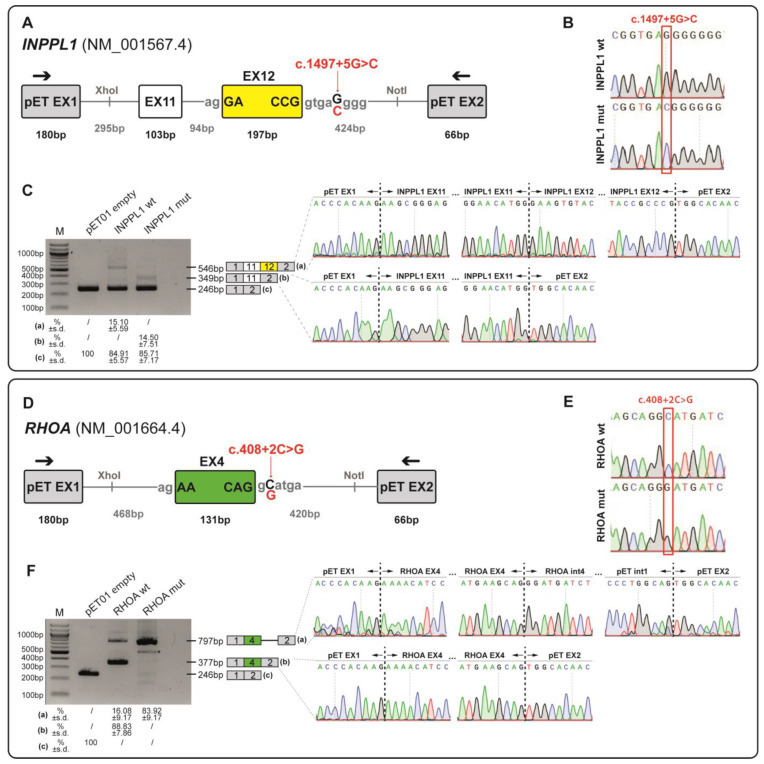
Minigene splicing assays for functional validation of *INPPL1* and *RHOA* variants. (**A**–**D**) Schematic representation of the genomic fragments subcloned into the XhoI and NotI restriction sites of the pET01 vector. The wild-type (wt) or mutated (mut) minigenes contain specific exon (*INNPL1* exon 12—yellow box; *RHOA* exon 4—green box) flanked by ~200 bp of their natural intronic sequences (thick grey lines). The variants identified in each gene are highlighted in red. The primers used for cDNA amplification are within the pET01 exons 1 and 2 (black arrows). (**B**–**E**) Sequencing electropherogram of wt and mut minigenes to confirm the cloning quality and the presence of the reference or the NM_001567.4(*INPPL1*):c.1497+5G>C and NM_001664.4(*RHOA*):c.408+2C>G variants in the wt and mut minigenes, respectively. (**C**–**F**) RT-PCR analysis of HEK293T cells transfected with empty pET01, wt and mut vectors. Sequencing chromatograms confirmed the identity of the bands. Numbers below the gel images represent isoform ratios expressed as mean (percentage, %) ± standard deviation (s.d.) of three independent experiments. *, aspecific band. M, marker 100 bp DNA Ladder (New England Biolabs, Ipswich, MA, USA).

The *INPPL1* variant NM_001567.4:c.1497+5G>C is localised five bases downstream of the exon 12—intron 12 boundary and is predicted by Splice AI to disrupt the canonical donor splice site of exon 12 (donor loss: 0.6). Minigene splicing assay (Figure 2A–C) showed that while the wild-type minigene generated a product of 546bp, corresponding to the normal inclusion of exon 11 and exon 12, the minigene carrying the c.1497+5G>C variant generated a product of 349 bp corresponding to the skipping of exon 12 (Figure 2C). This variant altered the reading frame leading to a premature termination codon (PTC) at amino acid 484, p.(Gly434Aspfs*52).

#### 3.4.2. Minigene Splicing Assay of NM_001664.4(*RHOA*):c.408+2C>G Variant

The de novo *RHOA* variant NM_001664.4:c.408+2C>G is predicted by SpliceAI to affect the canonical donor splice site of intron 4 of the *RHOA* gene (donor loss: 0.82).

Minigene assay (Figure 2D–F) showed that the c.408+2C>G variant induced intron retention (797 bp) in the mutant minigene (Figure 2F), resulting in the formation of a PTC p.(Glu137Glyfs*2) and the production of a truncated protein.

## 4. Discussion

Foetal structural abnormalities, ranging from isolated minor anomalies to severe and multisystemic disorders, can be detected in approximately 3% of all pregnancies [15,17]. Identifying a genetic diagnosis in the prenatal setting is pivotal to offer couples a personalised counselling and enhance their autonomy in informed pregnancy-related decision-making and management. The challenge of identifying a molecular diagnosis in the prenatal setting is further enhanced by the pressing need to make a decision about continuation or termination of pregnancy in early pregnancy stages, as established by different laws in different countries worldwide. For instance, in Italy, termination of pregnancy is regulated under Law 194/1978. According to this law, voluntary interruption of pregnancy is allowed during the first 90 days of gestation; afterwards, termination is only possible if the pregnancy or childbirth entails a serious threat to the woman’s life or in presence of pathological processes that constitute a serious threat to the woman’s physical or mental health, such as those associated with abnormalities or malformations of the foetus. The Italian legislation also specifies that whenever it is possible that the foetus may be viable, any appropriate action to save their life should be undertaken. As a consequence, therapeutic termination of pregnancy can be performed up to the 22nd week of gestation, thus limiting the timing for secondary investigations and molecular analyses. In this context, after non-conclusive first-tier investigations (standard karyotype and/or CMA analysis), the foetal phenotype is the major driving factor for couples’ decision-making. In case of termination of pregnancy, it is therefore necessary to offer families additional *post-mortem* diagnostic tests, with the final goal of identifying a precise aetiology and molecular diagnosis. In this light, this study describes the effectiveness of an integrated diagnostic strategy which includes a detailed phenotypic characterisation combined with genomic analyses (i.e., WES). Through this approach it was possible to reach a significant diagnostic yield (26.7%), definitively comparable or even higher than that reported in the literature [3,16,17,19,54,55].

As expected, this integrated approach led to a higher diagnostic yield for foetuses presenting with multiple malformations, while a slightly lower yield was achieved for major single organ malformations. Among these a particularly high diagnostic rate was achieved in foetuses presenting urogenital malformations (50%). However, it has to be noted that only four foetuses belong to this category and two of them, who both carry a heterozygous novel likely pathogenic variant in the *GREB1L* gene, causative of Renal hypodysplasia/aplasia 3 (MIM: # 617805), were conceived by the same couple. The absence of a paternal sample to confirm parental inheritance highlights some of the challenges that have to be faced when performing genetic analyses in duo or even singleton cases. Indeed, in these circumstances, variant interpretation could be harder and the difficulties in providing a precise genetic diagnosis and accurate recurrence risks may pose great psychological burden on families. Furthermore, a high diagnostic yield was highlighted in foetuses with skeletal dysplasias (42.9%), in line with previous studies [5]. Among them, a noteworthy case is represented by a foetus presenting with long bones and ribs shortening and carrying a homozygous splicing variant in the *INPPL1* gene, associated with autosomal recessive Opsismodysplasia (MIM: # 258480). Despite the variant was previously classified as VUS, the results of the functional studies here described highlight its deleterious impact on *INPPL1* mRNA processing, allowing us to demonstrate its causative role and thus to reclassify it as likely pathogenic (ACMG/AMP criteria: PS3; PM2_supporting; PM3_supporting; PP3). Additionally, in one skeletal dysplasia case, only a maternally inherited variant was highlighted in the *TRIP11* gene, causative of autosomal recessive Achondrogenesis, type IA (MIM: # 200600). Despite the foetal phenotypic features may be consistent with those of this condition, we failed to identify the second mutated allele. This might be due to the intrinsic limits of WES analysis, which, for instance, is not able to identify exon-level CNVs or structural variants.

Two peculiar findings of our study are represented by the identification of a novel missense variant in the *ZNF292* gene and a splicing germline variant in the *RHOA* gene. The former is associated with Intellectual developmental disorder, autosomal dominant 64 (MIM: # 619188), a recently described condition that is characterised by developmental delay, intellectual disability with or without autism spectrum disorder and attention deficit–hyperactivity disorder, epilepsy, growth anomalies, tone abnormalities, ocular alterations, and unspecific dysmorphic features. Few of the previously reported patients presented brain anomalies upon MRI, including cerebellar and callosal anomalies, ventriculomegaly, and periventricular nodular heterotopia [40]. Interestingly, the identified foetus showed corpus callosum hypoplasia and dilated cerebral ventricles. Hypoplasia of the corpus callosum is a not-so-rare finding that is not considered specific of a single genetic disease and even though further studies are needed to gain insight into the molecular basis of *ZNF292*-related disorder, this is the first report of prenatally detected CNS anomalies in a foetus carrying a variant in the *ZNF292* gene. Few somatic missense variants in the *RHOA* gene have been so far associated with Ectodermal dysplasia with facial dysmorphism and acral, ocular, and brain anomalies (MIM: # 618727), a neuroectodermal disorder for which no prenatal phenotype has been described [43]. In this cohort, a novel splicing variant has been identified in a foetus presenting with congenital diaphragmatic hernia (CDH), facial dysmorphisms, and right foot sandal gap. Interestingly, even if CDH has not been reported in patients affected by Ectodermal dysplasia with facial dysmorphism and acral, ocular, and brain anomalies, animal models of CDH have been shown to present altered pulmonary RhoA expression, which may be linked to pulmonary hypertension [56]. Furthermore, a nonsense variant in the *RHOA* gene has been reported in the scientific literature as potentially linked to congenital heart disease [57]. In this study, we confirmed the impact of the identified *RHOA* variant on pre-mRNA splicing by a functional minigene assay. However, as literature data might suggest that different molecular mechanisms may underlie different *RHOA*-associated conditions, at present this finding should be considered a candidate association, and the identification of additional patients carrying loss-of-function variants in this gene will be mandatory to strengthen its validity.

A limitation of this study consists in the fact that, being based on a single Italian recruitment centre, it takes into consideration mainly foetuses born from families of Caucasian origins, thus potentially limiting the generalisability of the findings to broader and more diverse populations. However, an advantage of a single-centre study consists of the higher accuracy of data quality control, which minimises missing data. Overall, the present study highlights the diagnostic utility of an integrated clinical and molecular approach to achieve a precise genetic diagnosis for structural foetal abnormalities, thus providing families with precise recurrence risk estimations and detailed options about future pregnancies. Additionally, it is possible to underline how minigene assays provide a feasible way to assess the impact of splicing variants on pre-mRNA transcripts, especially when isolation of RNA and analysis of mRNA splicing from the original source is technically difficult or not possible.

In conclusion, despite the recent enormous breakthroughs in genomic medicine, the majority of foetal malformations still remain without an aetiologic diagnosis. In this light, prenatal genetic counselling is imperative to help couples to make informed decisions and only a multidisciplinary team of experts in foetal medicine and led by a Medical Geneticist can provide a comprehensive analysis of both the clinical and molecular picture even in the most complex cases. Moreover, this approach, once properly planned, could be carried out in few weeks to give couples an answer before legal deadlines for termination of pregnancies (in many countries between 22 and 24 weeks of gestation). Overall, a systematic implementation of the presented strategy in clinical routine could be crucial to foster the discovery of new genes involved in embryonic and foetal development.

## Figures and Tables

**Table 1:** Phenotypic classification of the enrolled foetuses. Diagnostic group: main diagnostic category according to the presence of major malformations of a single organ or multiple anomalies, including facial dysmorphisms. Phenotypic group: specific anomalies divided by affected organ or multisystemic condition. Total number: total number of foetuses belonging to each category; percentage is calculated over the entire cohort of 86 foetuses. Males—*n* (%): number of male foetuses identified in each category; percentage is indicated in brackets. Females—*n* (%): number of female foetuses identified in each category; percentage is indicated in brackets. *p*-value: a binomial test was performed to evaluate the difference in frequency of malformations between males and females in each phenotypic group. The statistical significance was set to *p*-value < 0.05, and the analyses were performed with R version 4.1.2 (R Foundation for Statistical Computing, Vienna, Austria).

Diagnostic Group	Phenotypic Group	Total Number (%)	Males—*n* (%)	Females—*n* (%)	*p*-Value
Single major malformation	CNS anomalies	21 (24.4)	11 (52.4)	10 (47.6)	0.52
Single major malformation	Skeletal anomalies	7 (8.1)	3 (42.9)	4 (57.1)	0.43
Single major malformation	Cardiac anomalies	5 (5.8)	3 (60)	2 (40)	0.60
Single major malformation	Urogenital anomalies	4 (4.7)	3 (75)	1 (25)	0.75
Single major malformation	Fluid accumulation	5 (5.8)	3 (60)	2 (40)	0.60
Multiple malformations	Multisystemic condition	44 (51.2)	28 (63.6)	16 (36.4)	0.64

## Data Availability

All phenotypic data presented in this study are included in this manuscript and its Supplementary Information Files. Sequencing data are not publicly available due to privacy restrictions and are available from the corresponding author upon reasonable request.

## Supplementary Materials

The following supporting information can be downloaded at https://www.mdpi.com/article/10.3390/genes16101167/s1, Figure S1: Causes of previous foetal or newborn loss; Table S1: Primer list; Table S2: Detailed phenotypic features of all foetuses with positive WES result; Table S3: Detailed phenotypic features of all foetuses with negative WES result.

## Abbreviations

The following abbreviations are used in this manuscript:

ACMG/AMP	American College of Medical Genetics/Association for Molecular Pathology
AD	Autosomal Dominant
AR	Autosomal Recessive
CMA	Chromosomal Microarray
CNS	Central nervous system
CNV	Copy Number Variant
COMP HET	Compound Heterozygous
CT	Computed Tomography
DMEM	Dulbecco’s Modified Eagle’s Medium
HEK	Human Embryonic Kidney cells
HET	Heterozygous
HGMD	Human Gene Mutation Database
HGVS	Human Genome Variation Society
HOM	Homozygous
HPO	Human Phenotype Ontology
MIM	Mendelian Inheritance in Man
MRI	Magnetic Resonance Imaging
MUT	Mutated
NGS	Next Generation Sequencing
NT	Nuchal Translucency
QF-PCR	Quantitative Fluorescence Polymerase Chain Reaction
RT-PCR	Reverse Transcription Polymerase Chain Reaction
SNP	Single Nucleotide Polymorphism
VUS	Variants of Uncertain Significance
WES	Whole-Exome Sequencing
WT	Wild-Type
XL	X-linked
XLD	X-linked Dominant
