# Supplementary material for: Beyond the Curtains: Identification of the Genetic Cause of Foetal Developmental Abnormalities Through the Application of Molecular Autopsy
Source: Genes (Basel). 2025 Oct 2;16(10):1167. doi: 10.3390/genes16101167 (PMC12562548; doi:10.3390/genes16101167)
Supplement: Supplementary file 1 [file genes-16-01167-s001.zip › Supplementary Figure S1.pdf]

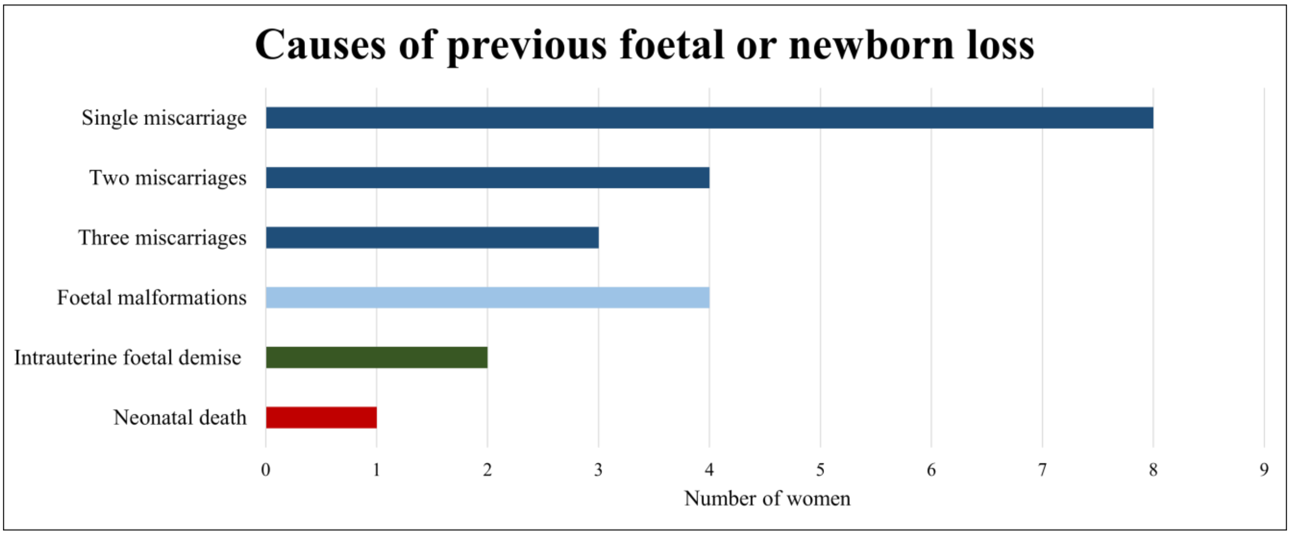

Supplementary Figure S1. Causes of previous foetal or newborn loss. Spontaneous first trimester miscarriages were frequently reported in anamnesis: eight women (9.3%) experienced a single miscarriage, while four (4.7%) and three (3.5%) of them experienced two and three previous miscarriages, respectively. Additionally, four women (4.7%) reported one or two previous terminations of pregnancy due to foetal malformations, two (2.3%) reported an intrauterine foetal demise during the second and third trimester of pregnancy, respectively, and one (1.2%) reported a previous neonatal death.
