# Supplementary material for: Beyond the Curtains: Identification of the Genetic Cause of Foetal Developmental Abnormalities Through the Application of Molecular Autopsy
Source: Genes (Basel). 2025 Oct 2;16(10):1167. doi: 10.3390/genes16101167 (PMC12562548; doi:10.3390/genes16101167)
Supplement: Supplementary file 1 [file genes-16-01167-s001.zip › Supplementary_Table_S1.pdf]

Name	Sequence 5’- -3’	Purpose
INPPL1-gDNA_F	CGGGCCCCCCCTCGAGCTTCCTGGATGCCTACCCG	Amplification for cloning
INPPL1-gDNA_R	ACCGCGGTGGCGGCCGCGATATGCTCAGGGACCAGAG	
RHOA-gDNA_F	CGGGCCCCCCCTCGAGACCACTCTTGGCTGGACTGAG	Amplification for cloning
RHOA-gDNA_R	ACCGCGGTGGCGGCCGCAGAGGGGCTTCTGAGCCTC	
ARID1B-gDNA-F	CGGGCCCCCCCTCGAGAATGGGTGTTCCCATCCCT	Amplification for cloning
ARID1B-gDNA-R	ACCGCGGTGGCGGCCGCTCCCAGGAGTACTTTCTAGCA	
pET01-EX1_RT-PCR_F	GATCGATCCGCTTCCTGCCCC	pET01 cDNA amplification
pET01-EX2_RT-PCR_R	CTGCCGGGCCACCTCCAGTGCC	
pET01-EX1_SEQ_F	GGATTCTTCTACACACCC	pET01 sequencing primers
pET01-EX2_SEQ_R	TCCACCCAGCTCCAGTTG	

Supplementary Table S1. Primer list. List of primers used in the study. Primers for cloning were designed using the Takara Bio primer design tool (https://www.takarabio.com/learning-centers/cloning/primer-design-and-other-tools). Restriction enzyme sites to subclone the fragments in the XhoI/NotI pET01-digested vector are underlined.
